# Medical Teaching in America

**Published:** 1899-01

**Authors:** 


### MEDICAL TEACHING IN AMERICA.

An American physician, writing to the *Clinical Review* from Berlin of medical affairs, and particularly of the inferior position of American graduates as compared with those of Germany,—due mainly, of course, to their longer courses and greater thoroughness,—says: "Although America now is fast approaching the foreign standard of excellence and thoroughness in medical education, it will be some time before the slow-witted Teuton will admit that anything American is as good as the German article; but the quickest way to teach him the supremacy of America is not to keep sending over here our medical men to study in his schools after they have finished with ours. Give our doctors better facilities and advantages in our large cities. * * * Let our professors become famous by original research and experimentation, by clever operating and brilliant diagnosis, and we will not see our students going abroad to finish their education and be snubbed by their German cousins."—*Maryland Medical Journal.*

